# Source Apportionment of Particulate Matter in a Metal Workshop

**DOI:** 10.3390/ijerph21060768

**Published:** 2024-06-13

**Authors:** Antonella Buljat, Marija Čargonja, Darko Mekterović

**Affiliations:** Faculty of Physics, University of Rijeka, Radmile Matejčić 2, 51000 Rijeka, Croatia; buljatantonella@gmail.com (A.B.); mcargonja@uniri.hr (M.Č.)

**Keywords:** air pollution, welding, machining, X-ray fluorescence, elemental composition, positive matrix factorization

## Abstract

Metal workshops are workplaces with the substantial production of particulate matter (PM) with high metal content, which poses a significant health risk to workers. The PM produced by different metal processing techniques differs considerably in its elemental composition and size distribution and therefore poses different health risks. In some previous studies, the pollution sources were isolated under controlled conditions, while, in this study, we present a valuable alternative to characterize the pollution sources that can be applied to real working environments. Fine PM was sampled in five units (partially specializing in different techniques) of the same workshop. A total of 53 samples were collected with a temporal resolution of 30 min and 1 h. The mass concentrations were determined gravimetrically, and the elemental analysis, in which the concentrations of 14 elements were determined, was carried out using the X-ray fluorescence technique. Five sources of pollution were identified: background, steel grinding, metal active gas welding, tungsten inert gas welding, and machining. The sources were identified by positive matrix factorization, a statistical method for source apportionment. The identified sources corresponded well with the work activities in the workshop and with the actual sources described in previous studies. It is shown that positive matrix factorization can be a valuable tool for the identification and characterization of indoor sources.

## 1. Introduction

Particulate matter (PM) is a mixture of solid particles and liquid droplets suspended in the atmosphere and is considered one of the most important air pollutants. Fine particles, usually defined as particles with an aerodynamic diameter of less than 2.5 µm (PM_2.5_), are of particular interest as they can easily enter the human respiratory system and possibly cause adverse health issues [1].

Many studies have been conducted to monitor outdoor PM exposure, but since many individuals spend most of their time indoors, monitoring indoor PM exposure is of particular importance. Certain workplaces are of particular interest since workers are occupationally exposed to high levels of PM (among other health risks). Metal workshops, where metal parts (mostly steel) are welded, cut, polished, etc., are places where significant amounts of metal particles are generated [2,3,4,5].

The particles produced during welding usually have a diameter of less than 2.5 µm and often even less than 1 µm [6,7], which is why they enter the human respiratory tract relatively easily. Metals, which are usually essential components of these particles, can then accumulate in certain tissues and potentially cause health issues [4,8,9,10,11,12]. The International Agency for Research on Cancer (IARC) has classified welding fumes as carcinogenic to humans (Group 1) [13].

Welding is the joining of metal parts that have been softened or rendered liquid by intense heat—in most cases, by an electric arc. The arc is created between the base metal and the welding electrode or wire. In gas metal arc welding (GMAW), an arc is created between a consumable electrode and the metal parts, which is shielded with either an inert gas (metal inert gas welding, MIG) or an active gas (metal active gas welding, MAG). In tungsten inert gas (TIG) welding, a non-consumable tungsten electrode is used to form the electric arc, while the filler metal is used to join the metal parts [14]. In any type of welding, fumes are generated from the heated and partially volatilized metal parts (base metal, electrode filler material), as well as from the electrode coating, shielding gases, fluxes, and paint surface coatings [8]. The vaporized metals react with the air and produce metal oxides, which condense and form particles that are mainly of respirable size [8]. Machining, on the other hand, is a mechanical process in which the excess of the metal is removed in order to obtain the desired shape and form. During the process, the metal is not significantly heated, but particles can be produced by mechanical removal and from the metalworking fluid used to reduce friction [15].

The composition of the particles generated by the various welding techniques depends on many factors, including the composition of the welding materials and shielding gases, the temperature, the material coatings and paints, etc. [6,16]. In addition, the final particle concentration depends on the size of the workshop, its ventilation, and the activities carried out in it.

The characterization of the elements is of crucial importance for the assessment of the health effects of particulate matter, as the toxicity of particulate matter strongly depends on its composition. Moreover, toxic effects are caused not only by individual elements but also by specific combinations of elements, so it is advisable to assess the contributions of different pollution sources with characteristic combinations of elements [17,18,19,20]. For example, high concentrations of Mn are neurotoxic, and Fe and Mn have been shown to share some important transporters in respiratory, intestinal, or brain cells. As Mn and Fe are often produced simultaneously during welding, high concentrations of Fe can further increase the uptake of Mn into the body [21]. Several recent studies have investigated welding as an important source of particulate matter pollution [5,12,22,23,24,25,26,27,28,29,30,31]. For our research, of particular interest are studies that have investigated specific types of welding (such as MIG or TIG), different welding conditions (such as different voltages, electrodes, or temperatures), and different base materials, which have previously been studied by measuring the concentrations of one to 18 different metals. These studies were conducted either under controlled/semi-controlled conditions [7,32,33,34,35,36] or in real work environments [3,5,22,23,29,30,31,32,37,38,39,40,41,42,43]. Under controlled/semi-controlled conditions, a single type of welding could be completely isolated from the others. On the other hand, studies in real work environments are more relevant to assessing potential health hazards, but isolating specific welding techniques is more challenging. While some broad conclusions can be reached, the elemental composition obtained in these studies varies considerably.

In our study, we adopted a different approach that, to our knowledge, has not been attempted before. The characterization of the different types of welding/metal processing in a real working environment was achieved by source apportionment, a standard procedure for the identification of pollution sources in outdoor air [44]. The particulate matter was sampled in five units of a single metal workshop, each of which partially specialized in a certain metal processing technique, which may be helpful in interpreting the pollution sources identified by source apportionment.

## 2. Materials and Methods

PM_2.5_ was sampled in 5 units of a single metal workshop in Rijeka, Croatia. The units specialized in one or several processing techniques. The characteristics of each unit are listed in Table 1, with the materials and techniques declared by the shift supervisors. Nevertheless, care should be taken with the techniques performed, because, in the real working environment, in addition to welding, grinding is often performed to prepare the base material for welding and later to flatten the weld.

Sampling was performed with a cyclone sampler based on the ANSTO ASP sampler [45] with a cut-off size of 2.5 µm. The sampler was positioned in close proximity to the techniques performed, usually a few meters from the first working station, but other working stations were also located at different positions inside the unit. The sampler inlet was positioned at a 1.7 m height, while the height of each unit was 10 m. The hourly or 30 min samples were collected on thin polytetrafluoroethylene (PTFE) membrane filters (*d* = 25 mm). A total of 53 samples were collected, out of which 47 were 30 min samples and 6 were hourly samples. The total mass concentration was determined gravimetrically using a Mettler Toledo XA105 dual-range balance (Greifensee, Switzerland). Each filter was weighed before and after sampling to determine the mass of the deposit. The filters were conditioned at 22 °C and 20% humidity (in a desiccator) prior to weighing. This procedure and the very low moisture absorption of PTFE enabled stable mass measurements.

The elemental analysis was carried out using the energy-dispersive X-ray fluorescence technique (ED-XRF). A low-power rhodium X-ray tube was used for excitation at 50 kV and 1 mA. The beam was collimated to 2 mm and directed perpendicularly to the sample. A total area of 8 mm^2^ was scanned on each filter to eliminate the effect of possible inhomogeneities in the samples. The characteristic X-rays were collected with a silicon drift detector positioned at an angle of 135° with respect to the incident beam. The resulting spectra were analyzed using the QXAS software [46] to determine the concentrations of S, Cl, K, Ca, Ti, Cr, Mn, Fe, Ni, Cu, Zn, Mo, Pb, and Bi.

The measurement uncertainties were estimated from the fitting errors (obtained with spectrum analysis software), the uncertainty of the system efficiency calibration, and the uncertainty of the volume measurement (obtained from a calibrated flowmeter during air sampling). The uncertainties were typically about 5–10% for major elements like Fe, Mn, and Zn, while they were around 20–40% for S, Cl, and Pb. For Mo and Bi, the uncertainties were around 50%, or sometimes even higher. The minimum detection limit for each element was determined from the typical background level during the fitting procedure. The values for the uncertainties and the minimum detection limits can be found in the Supplementary Materials, along with the concentration data (Tables S1–S3).

Source apportionment was carried out using positive matrix factorization (PMF) [47,48]. The EPA PMF 5.0 model was used for this purpose [49]. In this procedure, the measurement inputs are two matrices: the matrix *X* with the elemental and PM concentrations, and the matrix *U* with the corresponding measurement uncertainties, where the number of rows is the number of samples, *n* (53 in our case), and the number of columns is the number of species, *m* (14 elements plus PM_2.5_). The additional information that should be provided is the presumed number of pollution sources, *p* (in our case, these include different metal processing techniques). *X* can be written as

(1) X=G·F+E

where *F* is the matrix specifying the sources (matrix element *f_kj_* is the fraction of the *j*^th^ species in the *k*^th^ source), *G* is the matrix specifying the contribution of the source to the sample mass (matrix element *g_ik_* is the contribution of the *k*^th^ source in the *i*^th^ sample), and *E* is the matrix of the residuals. *G* and *F* are found by minimizing the objective function *Q*:

(2) $$Q=\sum_{i=1}^{n}\sum_{j=1}^{m}{\left[\frac{x_{ij}-\sum_{k=1}^{p}g_{ik}f_{kj}}{u_{ij}}\right]}^{2}$$

where *u_ij_* are the elements of the matrix *U*. In an ideal case, *G* and *F* should be strictly positive (hence, positive matrix factorization), but, in practice, this is usually relaxed so that no samples can have significantly negative source contributions. Two versions of *Q* are calculated: *Q*(true), calculated for all points, and *Q*(robust), calculated while excluding points with high residuals.

PM_2.5_ was treated as a total variable (all other elements contributed to it), while Pb and PM_2.5_ were treated as weak variables (which tripled the provided uncertainty). In situations where a certain element was not detected in a given sample, half of the minimum detection limit was used instead of the concentration.

A more detailed description of the model can be found elsewhere [47,48,49].

An optical particle counter (OPC) was used to measure the number concentrations of particles with an optical diameter of 0.3 μm–0.5 μm, 0.5 μm–1 μm, 1 μm–2.5 μm, 2.5 μm–5 μm, 5 μm–10 μm, and >10 μm. The measurements were carried out with a time resolution of 5 s.

## 3. Results

**Figure 1:**
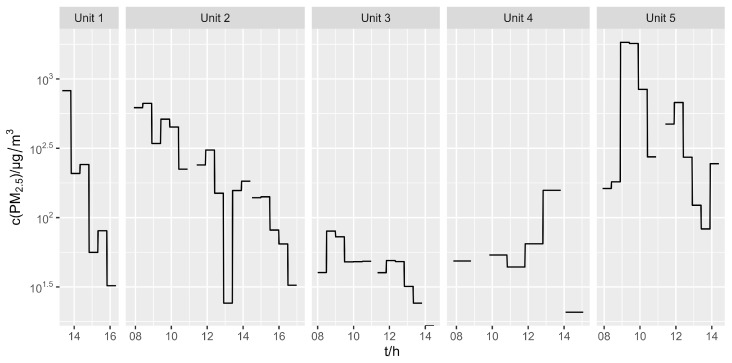
Time series of PM_2.5_ concentrations in 5 workshop units.

The PM_2.5_ concentrations are shown in Figure 1, while the time series for all elements can be found in the Supplementary Materials (Figure S1a,b). All measured concentrations are also given in Table S1. The PM_2.5_ concentrations varied from approximately 20 to 1800 µg/m^3^, being the highest in Unit 5, where MAG welding, polishing, and cutting were performed. Units where TIG welding was carried out (Units 3 and 4) had the lowest PM_2.5_ concentrations.

**Figure 2:**
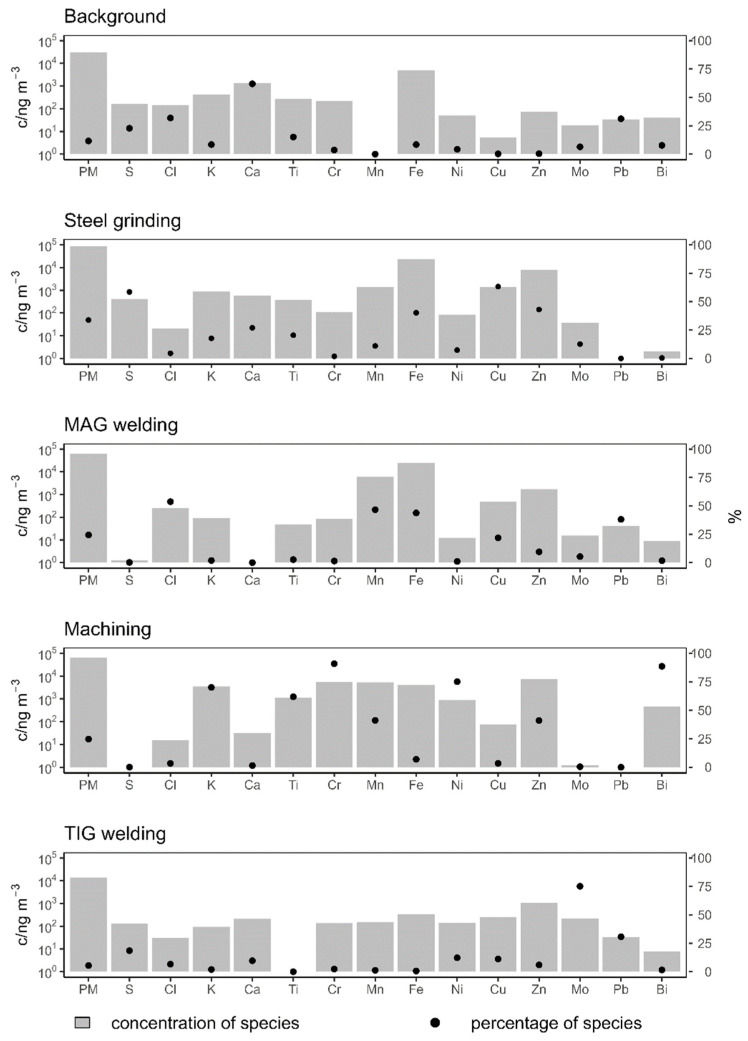
Elemental characterization of 5 pollution sources from the PMF analysis. Species concentrations (in ng m^−3^, left scale) are shown in grey columns, while the percentages of each species assigned to a source are shown by black dots (right scale).

**Figure 3:**
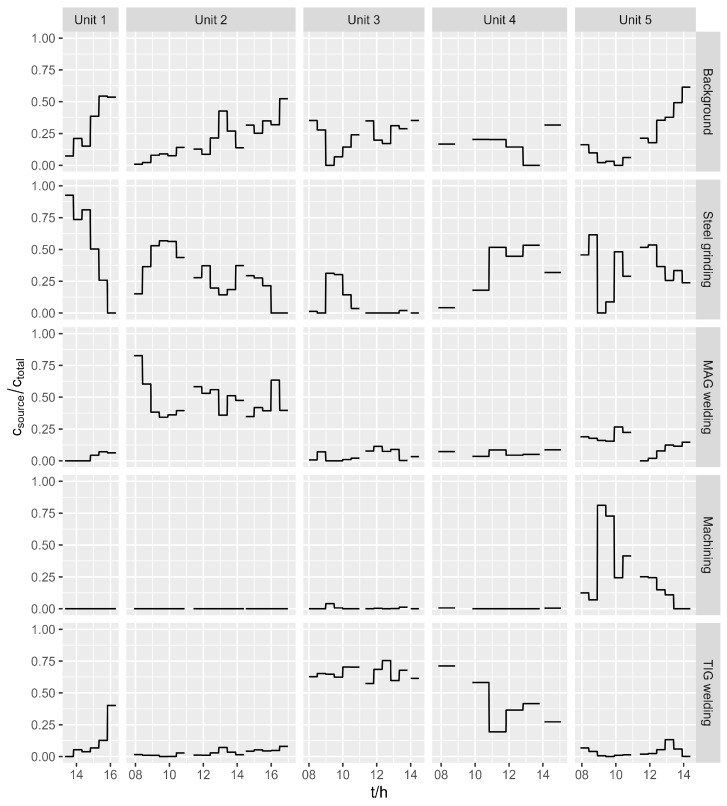
Portion of each pollution source in the total pollution. The concentration of the individual pollution source was normalized to the sum of all sources.

Positive matrix factorization was then performed with the concentrations of PM_2.5_ and 14 elements. A solution with five pollution sources was chosen. The characteristic fingerprints of the sources are shown in Figure 2, where the elemental concentrations are represented as constituents of the sources (grey columns), as estimated with PMF. Moreover, the percentages of occurrence for different elements in the sources are presented with black dots. The time series of the relative contributions of each source to the total pollution (source concentrations normalized to the sum of all sources) are shown in Figure 3.

The first source is characterized by high contributions of Ca and Fe and low contributions of Mn, Ni, and Cu and by their continuous presence in all units. For these reasons, this source was named ‘background’ and was considered as a combination of particles from the outside and particles already present in the workshop but resuspended due to the work activities.

The second source was characterized by high concentrations of Fe, Zn, Cu, and Mn. It was mainly present in Units 1, 2, 4, and 5 and was named ‘steel grinding’. It was a predominant source in Unit 1, where no welding was performed, as well as a significant source in units where grinding and polishing were declared in addition to welding, making it a marker for the cutting, grinding, or polishing of the base material. Ideally, the metal is ground in a separate room before welding, but, very often, grinding is also performed in parallel with welding for the final adjustment of the weld, so it is possible that some grinding was performed in each individual unit.

The third source was characterized by high Fe and Mn concentrations, with lower Zn concentrations than in the second source. It was a dominant source in Unit 2 and also present in Unit 5, where the MAG welding of mild steel was the predominant activity, so it was assumed that this source represented ‘MAG welding’.

The fourth source was characterized by high concentrations of K, Ti, Cr, Mn, Fe, Ni, Zn, and Bi, which was almost exclusively present in this source, and with a presence in Unit 5 only. Several techniques were reported for Unit 5, but no specific materials were reported for these techniques. We presume that different free machining steels were used, which often contain Bi for better mechanical properties during machining [50]. In addition to steel, other materials, such as Wood’s metal (which also contains Bi) or zinc alloys, are also commonly used for machining. For these reasons, this source was named ‘machining’.

The last source was characterized by high concentrations of Zn and the fact that Mo was almost exclusively present in this source. It was therefore named ‘TIG welding’, as a wire with Mo is used for this activity. The concentrations of Cr, Mn, Fe, Ni, and Cu were also high here, although not as high as in other sources. This source was a predominant source in Units 3 and 4.

**Figure 4:**
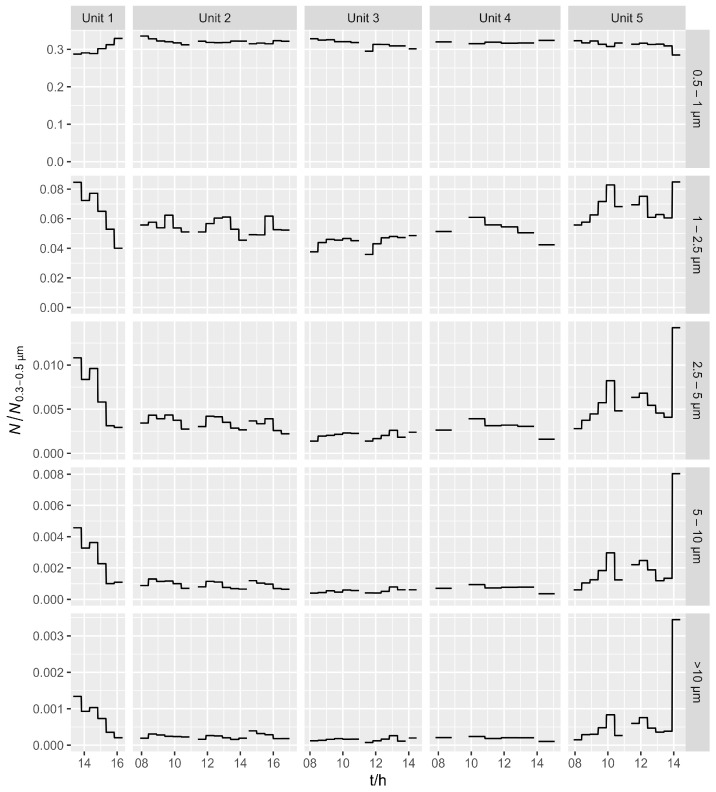
Number concentrations for five size ranges, measured by OPC, normalized to the concentrations of the smallest range (0.3–0.5 µm).

Figure 4 shows a time series of the number concentrations for five size ranges measured by OPC, all normalized to the number concentrations of the smallest size range (0.3–0.5 µm) in each of the workshop units. For the 0.5–1 µm size range, there is no obvious difference between the units, while, for the larger particle sizes, it is clear that Units 1 and 5 have a higher fraction of larger particles (compared to the other units). In general, larger particles are more often produced by non-hot processes such as grinding, cutting, or polishing, so it can be assumed that Units 1 and 5 had a higher share of these processes.

**Figure 5:**
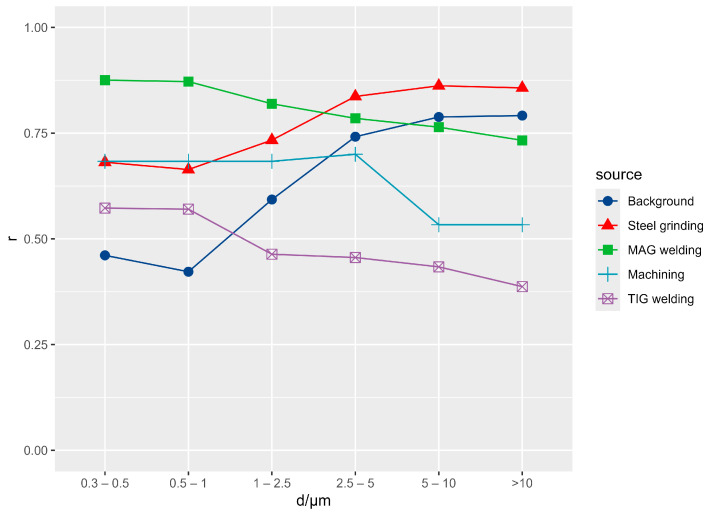
Spearman’s correlation coefficients between pollution sources and number concentrations for each size range. Only samples with at least 10% of the total pollution were selected for this calculation.

The size distribution for each pollution source was estimated using the method proposed by Mazzei et al. [51]. In contrast to this study, where Pearson’s correlation coefficient was used, Spearman’s correlation coefficient was chosen because the relationship between optical and gravimetric measurements is not linear, especially at high concentrations, where we observed a form of saturation. The results are shown in Figure 5, which shows the Spearman correlation between different optical diameters (averaged over a time interval of 30 min or 1 h, depending on the sample type) and different sources. When calculating the correlation coefficients, we only used samples where the source had a contribution of at least 10% of the sum of all sources, in order to select the data that were more representative of the source and to eliminate the samples with negative source contributions. The sensitivity to the choice of the threshold level was checked by varying the threshold from 5 to 15%. The results were very stable for background and steel grinding. For MAG and TIG welding, we always observed the same trend where the correlation coefficients decreased with the particle size but changed significantly in absolute value. The results for machining should not be considered reliable due to the small data set (the source of machining was exclusively present in Unit 5). The MAG welding and TIG welding sources show a decrease in the correlation coefficient at larger optical sizes, which means that these sources have higher contributions of smaller particles than the other sources. This is the general characteristic of particles produced by welding, as found in previous studies [2,52,53]. The steel grinding and background sources, on the other hand, show the opposite pattern and are more strongly correlated with the larger particles. The steel grinding source corresponds to a process without the heating of the material, so that the proportion of larger particles is greater than in welding. The background source contains particles from the outside as well as particles resuspended from previous activities.

## 4. Discussion

The main input in the PMF analysis is the number of pollution sources. There is no simple recommended criterion for the choice of this number, as the assumption that there is a small number of stable sources is rarely strictly satisfied in practice. Increasing the number of presumed sources improves the agreement between the prediction and measurement, but the sources themselves become unstable and more difficult to interpret. Therefore, the focus should be on the robust aspects of the solution when varying the number of pollution sources and other inputs. We performed the analysis with four to six sources, checked the effect of removing certain species or classifying them as weak, and removed some samples as outliers. Moving from four to six sources, we observed that background, TIG welding, and especially machining were stable. Only the fourth source was initially split into MAG welding and steel grinding, and, later, MAG welding was split into two sources with similar characteristics.

The comparison with the previously reported data was performed by calculating the ratio of metal concentrations (namely Cr, Mn, Ni, and Cu) and Fe concentrations, as Fe was a predominant element in most of the samples. Only studies that reported analyses for the same welding techniques and materials as in the present study were used for this comparison.

The comparison with our results for MAG welding was performed with studies that analyzed particles produced by the gas metal arc welding of mild steel [3,4,6,34,39]. Mn was one of the most frequently investigated elements in these studies. In nine previous studies, the Mn/Fe ratio was measured to be between 7 and 33% (with a median of 19%), while the ratio in the current study was 24%. The Cr/Fe ratio was 0.08% and 0.4% in two studies, while it was 0.3% in the present study. The Ni/Fe ratio was 0.1‰, 5‰, and 20‰ in three studies, while it was 0.5‰ in the present study.

Only one study was found that included the TIG welding of stainless steel [3], in which only the Fe and Mn concentrations were measured and the Mn/Fe ratio was 44%, while it was 46% in the present study.

Two studies were found for the comparison with grinding. Karlsen et al. (1992) [32] measured a Mn/Fe ratio of 3 to 7%, a Cr/Fe ratio of 5%, and a Ni/Fe ratio of 8 to 13% under three different conditions. Berlinger et al. (2019) [6] measured a Mn/Fe ratio of 32%, a Cr/Fe ratio of 3%, and a Ni/Fe ratio of 1%. Our results were 6% for the Mn/Fe ratio, 0.5% for the Cr/Fe ratio, and 0.4% for the Ni/Fe ratio.

Since the ranges of the ratios reported in the literature are relatively large (as expected from studies under different conditions), our results for the identified sources are in good agreement with the actual sources described in previous studies.

While we had expected, or at least hoped, to identify grinding and MAG and TIG welding, as these were the reported activities, the identification of the machining source was unexpected. Its most characteristic feature is the presence of Bi, which is not found in steels used for welding and which we did not observe in Units 1–4. However, this source is not only determined by the presence of Bi. In fact, this was our most stable finding, because the time series of this source almost did not change at all when Bi was excluded from the analysis. This is because several elements (most notably K, Cr, and Mn) in Unit 5 were strongly correlated with Bi. We hypothesize that the presence of K originates from potassium soap, which is sometimes used as an emulsifier in metalworking fluids during machining [54]. Indeed, K is sometimes used as a marker for the detection of airborne metalworking fluids [55]. It can therefore be assumed that machining operations (turning, milling, etc.) were carried out in Unit 5 and the adjacent mechanical unit.

In Units 3 and 4, stainless steel was reported as the predominant material used. We assume that type 1.4404 steel (commonly used in shipbuilding [56]) and zinc-plated steel (mostly pipes) were used. When working with zinc-plated steel, the surface must be ground before welding and afterwards to flatten the weld seam. This process releases large amounts of Zn in the form of particles, so this is most likely the reason for the high Zn content in the source of TIG welding.

The interpretation of the sources identified by PMF should be performed with some caution—the names given to the sources represent our best estimates of the actual pollution sources. This estimate is based on the workshop’s work plan and materials list; the elemental composition, which was also compared to previous studies; and the particle size distribution. However, we believe that this identification has successfully described the most important aspects of the sources.

Previous studies have shown that coarse particles (with a diameter higher than 2.5 µm) are deposited mainly by impaction in the tracheobronchial region and fine particles (with a diameter of less than 2.5 µm) are deposited mainly by sedimentation and diffusion in the pulmonary region [57]. However, since the respiratory and cardiovascular effects of inhaled particles depend on the total particle surface area, ultrafine particles (with a diameter of less than 0.1 µm) are considered to be more toxicologically relevant than larger particles [58]. In the present study, however, only particles with a diameter of more than 0.3 µm were analyzed.

Since this study was only conducted for one week and the employees indicated that the workload varied from week to week (and was sometimes much higher than in this study), these results should not be used to assess the overall pollution from a particular technique. The results may be valuable to characterize the specific fingerprint of each pollution source, as it can be assumed that the techniques do not change significantly (although some other materials and processes could be introduced). Of course, the characterization itself would also be more reliable if more samples were available, so further studies are strongly recommended. Since the particle size distribution is another important parameter in the characterization of sources, such a study would also benefit from measurements of lower particle sizes than in the present study.

## 5. Conclusions

The characterization of pollution sources was carried out in a real working environment, in a metal workshop where the different metalworking techniques were not completely isolated. The pollution sources were identified and characterized using positive matrix factorization, a statistical technique commonly used in air quality studies. This was the first time that it was used for an indoor working environment. Five pollution sources were identified: background, steel grinding, MAG welding, TIG welding, and machining. The previous literature included the characterization of pollution from isolated welding techniques on the same materials as in the present study. This literature was sparse but in good agreement with the present study. Although the identification of the pollutant sources can only be considered approximate, we believe that it was mostly successful as it related well to the work schedule and the materials and techniques used in the workshop. It can, therefore, be concluded that this statistical method can be a valuable tool for the monitoring of indoor air quality.

## Figures and Tables

**Table 1:** Characteristics of 5 units of the single metal workshop included in the study.

Workshop Unit	1	2	3	4	5
**Unit area/m^2^**	400	800	800	800	800
**Technique**	grinding	MAG welding *	TIG welding **	TIG welding **, polishing, grinder cutting	MAG welding *, polishing, grinder cutting, gas cutting
**Materials**	steel	steel	stainless steel	stainless steel	steel, stainless steel
**Electrode/wire**	-	steel electrode 0.08% C 0.5% Cu 0.85% Si 1.5% Mn	stainless steel wire 0.03% C 18% Cr 11% Ni 2% Mo 2% Mn 0.65% Si 0.75% Cu	stainless steel wire 0.03% C 18% Cr 11% Ni 2% Mo 2% Mn 0.65% Si 0.75% Cu	steel electrode 0.08% C 0.5% Cu 0.85% Si 1.5% Mn stainless steel electrode 0.03% C 0.7% Si 1.4% Mn 23% Cr 12.5% Ni
**Sample type**	30 min	30 min	30 min	1 h	30 min
**Number of samples**	6	17	12	6	12
**Note**	separated from Unit 2 by door	separated from Unit 1 by door	joined with Unit 4	joined with Unit 3	joined in part with a mechanical unit

* MAG welding—metal active gas welding. ** TIG welding—tungsten inert gas welding.

## Data Availability

The data are contained within the Supplementary Materials.

## Supplementary Materials

The following supporting information can be downloaded at: https://www.mdpi.com/article/10.3390/ijerph21060768/s1, Figure S1a. Time series of PM_2.5_ and elemental concentrations in five different units of a single metal workshop; Figure S1b. Time series of elemental concentrations in five different units of a single metal workshop; Table S1. PM_2.5_ and elemental concentrations measured in five units of a single metal workshop included in the study. All concentrations are given in µg/m^3^. Table S2. Estimated uncertainties for PM_2.5_ and elemental concentrations measured in five units of a single metal workshop included in the study. All uncertainties are given in µg/m^3^. Table S3. Minimum detection limits for PM_2.5_ and elemental concentrations in µg/m^3^.
