# Supplementary material for: Source Apportionment of Particulate Matter in a Metal Workshop
Source: Int J Environ Res Public Health. 2024 Jun 13;21(6):768. doi: 10.3390/ijerph21060768 (PMC11203473; doi:10.3390/ijerph21060768)
Supplement: Supplementary file 1 [file ijerph-21-00768-s001.zip › ijerph-3034591-supplementary.pdf]

Source apportionment of particulate matter in a metal workshop:

Supplementary Materials

Antonella Buljat[1], Marija Čargonja[1], Darko Mekterović[1*]

[1] Faculty of Physics, University of Rijeka, Radmile Matejčić 2, 51000 Rijeka, Croatia

* Corresponding author

In this Supplementary Materials, all concentration data are listed in Table S1 and shown in Figures S1a-b. The times indicate the start and end of each sample. For technical reasons, occasional breaks were taken during sampling. All concentrations in Table S1 are rounded according to the estimated measurement uncertainty. Where concentrations were lower than the minimum detection limit (MDL), MDL/2 value is shown instead of the concentration. Measurement uncertainties are included in Table S2, while minimum detection limits are included in Table S3.

Table S1. $PM_{2.5}$ and elemental concentrations measured in five units of a single metal workshop included in the study. All concentrations are given in μg/m^3.

unit	time	$PM_{2.5}$	S	Cl	K	Ca	Ti	Cr	Mn	Fe	Ni	Cu	Zn	Mo	Pb	Bi
1	13:20 - 13:50	823	4.4	0.4	8.2	7.4	9.9	0.60	10.1	290	0.66	10.4	42	0.3	0.09	0.03*
	13:50 - 14:20	208	1.1	0.3	1.5	3.1	1.1	0.16	1.8	45	0.38	6.5	12.5	0.2	0.07	0.03*
	14:20 - 14:50	241	1.5	0.2	2.6	2.8	2.5	0.22	2.9	79	0.43	7.2	12.6	0.2	0.12	0.03*
	14:50 - 15:20	56	0.5	0.4	0.81	1.6	0.81	0.10	0.89	16.3	0.14	0.96	5.1	0.12	0.10	0.03*
	15:20 - 15:50	80	0.18	0.3	0.31	0.99	0.25	0.08	0.37	5.5	0.07	0.37	1.44	0.09	0.08	0.03*
	15:50 - 16:20	32	0.20	0.07	0.09	0.73	0.18	0.10	0.12	3.0	0.10	0.16	0.71	0.1	0.05	0.03*
2	7:55 - 8:25	621	0.6	1.8	1.1	1.1	0.64	0.27	48	252	0.20	6.3	28	0.28	0.26	0.03*
	8:25 - 8:55	667	1.6	1.3	2.0	2.3	2.4	0.30	43	249	0.29	6.7	34	0.3	0.20	0.03*
	8:55 - 9:25	343	1.3	2.0	3.3	2.3	1.9	0.23	19	123	0.23	3.7	29	0.18	0.17	0.03*
	9:25 - 9:55	513	1.6	1.1	9.6	8.3	1.7	0.21	26	188	0.26	4.4	56	0.2	0.13	0.03*
	9:55 - 10:25	450	1.6	0.8	2.9	3.1	1.3	0.24	16.6	173	0.22	4.1	53	0.19	0.16	0.03*
	10:25 - 10:55	224	0.8	0.6	1.2	1.9	0.56	0.14	7.2	87	0.14	2.0	29	0.19	0.25	0.03*
	11:25 - 11:55	240	0.6	1.3	1.3	2.0	0.72	0.12	17.7	94	0.11	2.0	12.7	0.13	0.20	0.03*
	11:55 - 12:25	307	0.6	0.8	3.8	1.8	1.0	0.14	17.0	98	0.22	3.3	13.3	0.15	0.28	0.03*
	12:25 - 12:55	150	0.3	0.6	3.4	2.7	0.71	0.12	10.7	48	0.10	1.23	6.9	0.14	0.16	0.03*
	12:55 - 13:25	24	0.21	0.4	2.6	3.0	0.32	0.09	3.5	13.8	0.09	0.48	2.3	0.11	0.12	0.03*
	13:25 - 13:55	157	0.6	0.4	2.6	2.2	0.42	0.10	8.9	54	0.13	1.9	3.1	0.12	0.18	0.03*
	13:55 - 14:25	183	0.5	0.6	1.2	1.6	0.9	0.13	11.6	63	0.11	1.72	16.0	0.12	0.18	0.03*
	14:30 - 15:00	139	0.3	0.6	1.4	8.1	0.46	0.09	3.8	28	0.09	0.96	6.6	0.17	0.16	0.03*
	15:00 - 15:30	141	0.4	0.6	1.4	6.6	0.27	0.09	4.4	29	0.09	1.18	5.7	0.16	0.13	0.03*
	15:30 - 16:00	81	0.4	0.3	1.3	1.3	0.39	0.09	4.2	22	0.08	0.95	2.2	0.10	0.14	0.03*
	16:00 - 16:30	65	0.3	0.4	1.7	0.90	0.63	0.07	7.3	24	0.05	0.48	0.77	0.11	0.09	0.03*
	16:30 - 17:00	33	0.21	0.2	0.80	1.4	0.34	0.09	3.4	12.0	0.07	0.23	0.55	0.11	0.08	0.03*
3	8:00 - 8:30	40	0.3	0.11	0.35	1.5	0.15	0.28	0.31	2.7	0.25	0.41	2.7	0.4	0.08	0.03*
	8:30 - 9:00	80	0.4	0.15	0.37	1.5	0.13	0.45	0.71	2.8	0.39	0.65	2.1	0.7	0.09	0.03*
	9:00 - 9:30	73	0.6	0.04	0.46	0.66	0.09	0.48	0.51	2.5	0.44	0.79	11.2	0.8	0.10	0.03*
	9:30 - 10:00	48	0.4	0.25	0.64	0.70	0.12	0.38	0.50	5.0	0.37	0.89	6.5	0.7	0.14	0.03*
	10:00 - 10:30	48	0.4	0.19	0.51	0.88	0.13	0.55	0.61	5.1	0.49	0.81	3.8	1.0	0.10	0.03*
	10:30 - 11:00	48	0.5	0.13	0.56	1.03	0.13	0.46	0.53	3.8	0.43	0.62	2.4	0.8	0.10	0.03*

	11:20 - 11:50	40	0.23	0.14	0.31	1.3	0.08	0.25	0.38	2.1	0.21	0.31	1.37	0.3	0.11	0.03*
	11:50 - 12:20	49	0.27	0.20	0.38	0.78	0.07	0.34	0.81	2.8	0.31	0.46	3.4	0.5	0.09	0.03*
	12:20 - 12:50	48	0.3	0.13	0.34	0.89	0.07	0.41	0.66	3.1	0.40	0.51	3.1	0.6	0.10	0.03*
	12:50 - 13:20	32	0.16	0.14	0.24	0.96	0.08	0.30	0.43	2.7	0.25	0.39	1.68	0.4	0.08	0.03*
	13:20 - 13:50	24	0.19	0.12	0.17	0.36	0.06	0.18	0.17	1.60	0.16	0.22	1.40	0.2	0.11	0.03*
	14:00 - 14:30	17	0.4	0.07	0.24	1.2	0.07	0.33	0.20	1.50	0.32	0.33	0.76	0.6	0.05	0.03*
**4**	7:50 - 8:50	49	1.1	0.11	0.39	0.82	0.10	0.80	0.72	6.2	0.95	0.91	2.0	2.0	0.068	0.03*
	9:50 - 10:50	54	0.7	0.18	0.40	1.01	0.11	0.29	0.56	10.0	0.30	0.68	4.7	0.7	0.067	0.03*
	10:50 - 11:50	44	0.47	0.17	0.39	0.61	0.10	0.097	0.63	8.2	0.10	0.81	6.2	0.11	0.071	0.03*
	11:50 - 12:50	65	0.58	0.13	0.59	1.05	0.15	0.26	0.89	10.4	0.24	1.38	5.4	0.30	0.094	0.03*
	12:50 - 13:50	157	1.1	0.23	2.0	1.9	0.32	0.83	2.9	30	0.68	3.9	36	1.0	0.22	0.03*
	14:05 - 15:05	21	0.30	0.08	0.26	0.53	0.05	0.086	0.30	2.2	0.07	0.30	2.4	0.05	0.057	0.03*
**5**	7:55 - 8:25	162	0.6	0.2	2.6	1.6	1.1	2	8.6	28	0.38	1.58	6.5	0.21	0.14	0.21
	8:25 - 8:55	181	0.9	0.3	2.3	1.8	1.1	1	7.6	50	0.37	2.6	24	0.20	0.21	0.17
	8:55 - 9:25	1838	0.3	1.8	86	2.7	24	120	148	190	15.9	4.1	248	0.30	0.39	11.2
	9:25 - 9:55	1800	1.0	1.7	68	4.3	23	128	132	265	21	6.1	241	0.15	0.44	9.4
	9:55 - 10:25	841	1.3	1.0	13.9	2.9	4.7	20	30	278	3.5	10.1	87	0.4	0.12	1.6
	10:25 - 10:55	274	0.6	0.5	7.9	1.4	2.5	11.3	15.4	60	1.74	2.0	37	0.12	0.19	0.96
	11:25 - 11:55	473	1.8	0.6	10.5	10.9	9.9	12.5	16.3	108	2.5	9.2	31	0.3	0.08	0.91
	11:55 - 12:25	676	3.2	0.8	15.7	11.0	5.4	18.5	26	184	3.6	8.9	38	0.5	0.10	1.3
	12:25 - 12:55	273	1.0	0.5	4.5	6.7	4.6	5.2	7.3	61	1.12	2.2	13.9	0.3	0.16	0.35
	12:55 - 13:25	123	0.8	0.3	2.0	2.7	1.0	1.66	3.8	30	0.65	1.11	4.9	0.3	0.13	0.17
	13:25 - 13:55	83	0.6	0.3	0.51	2.3	0.56	0.37	1.20	25	0.14	0.59	6.4	0.11	0.13	0.05
	13:55 - 14:25	245	1.1	0.6	3.0	16.4	1.7	0.52	4.3	52	0.21	0.84	13.3	0.13	0.16	0.12

* MDL/2 instead of the measured concentration

Table S2. Estimated uncertainties for $PM_{2.5}$ and elemental concentrations measured in five units of a single metal workshop included in the study. All uncertainties are given in μg/m^3.

unit	time	$PM_{2.5}$	S	Cl	K	Ca	Ti	Cr	Mn	Fe	Ni	Cu	Zn	Mo	Pb	Bi
1	13:20 - 13:50	43	0.5	0.1	0.4	0.4	0.5	0.04	0.003	0.5	15	0.05	0.5	2	0.2	0.04
	13:50 - 14:20	19	0.2	0.1	0.1	0.2	0.1	0.02	0.001	0.1	2	0.03	0.3	0.6	0.2	0.03
	14:20 - 14:50	19	0.2	0.1	0.2	0.2	0.2	0.02	0.001	0.2	4	0.03	0.4	0.6	0.1	0.03
	14:50 - 15:20	18	0.1	0.1	0.09	0.1	0.09	0.01	0.0006	0.06	0.8	0.02	0.05	0.3	0.08	0.03
	15:20 - 15:50	20	0.08	0.1	0.06	0.09	0.05	0.01	0.0005	0.04	0.3	0.02	0.03	0.08	0.08	0.03
	15:50 - 16:20	23	0.08	0.06	0.05	0.08	0.04	0.01	0.0006	0.02	0.2	0.02	0.03	0.04	0.1	0.02
2	7:55 - 8:25	26	0.2	0.3	0.1	0.1	0.09	0.02	0.002	2	13	0.03	0.3	1	0.07	0.04
	8:25 - 8:55	26	0.3	0.2	0.1	0.2	0.2	0.03	0.002	2	13	0.03	0.3	2	0.10	0.04
	8:55 - 9:25	26	0.2	0.3	0.2	0.2	0.2	0.02	0.001	1	6	0.03	0.2	1	0.07	0.03
	9:25 - 9:55	33	0.3	0.2	0.5	0.5	0.2	0.02	0.001	1	9	0.03	0.2	3	0.1	0.04
	9:55 - 10:25	22	0.3	0.2	0.2	0.2	0.1	0.02	0.001	0.8	9	0.03	0.2	3	0.08	0.04
	10:25 - 10:55	27	0.2	0.1	0.1	0.2	0.08	0.02	0.0008	0.4	4	0.03	0.1	1	0.06	0.02
	11:25 - 11:55	24	0.1	0.2	0.1	0.2	0.09	0.02	0.0007	0.9	5	0.03	0.1	0.6	0.05	0.02
	11:55 - 12:25	21	0.1	0.2	0.2	0.1	0.1	0.02	0.0008	0.9	5	0.03	0.2	0.7	0.05	0.02
	12:25 - 12:55	17	0.1	0.1	0.2	0.2	0.09	0.02	0.0007	0.5	2	0.02	0.07	0.3	0.06	0.02
	12:55 - 13:25	24	0.09	0.1	0.2	0.2	0.05	0.01	0.0006	0.2	0.7	0.02	0.03	0.1	0.06	0.02
	13:25 - 13:55	25	0.1	0.1	0.2	0.2	0.06	0.02	0.0006	0.5	3	0.03	0.10	0.2	0.05	0.02
	13:55 - 14:25	26	0.1	0.1	0.1	0.1	0.1	0.02	0.0008	0.6	3	0.03	0.09	0.8	0.05	0.02
	14:30 - 15:00	18	0.1	0.1	0.1	0.5	0.07	0.01	0.0006	0.2	1	0.02	0.05	0.3	0.07	0.02
	15:00 - 15:30	19	0.1	0.1	0.1	0.4	0.05	0.02	0.0005	0.2	1	0.02	0.06	0.3	0.08	0.02
	15:30 - 16:00	20	0.1	0.1	0.1	0.1	0.06	0.01	0.0006	0.2	1	0.02	0.05	0.1	0.05	0.02
	16:00 - 16:30	16	0.1	0.1	0.1	0.09	0.08	0.01	0.0005	0.4	1	0.02	0.03	0.05	0.07	0.02
	16:30 - 17:00	23	0.09	0.1	0.09	0.1	0.06	0.01	0.0006	0.2	0.6	0.02	0.03	0.04	0.09	0.02
3	8:00 - 8:30	18	0.10	0.08	0.06	0.1	0.03	0.02	0.002	0.03	0.1	0.03	0.03	0.1	0.3	0.02
	8:30 - 9:00	20	0.1	0.08	0.07	0.1	0.03	0.03	0.003	0.05	0.1	0.03	0.04	0.1	0.4	0.02
	9:00 - 9:30	24	0.1	0.07	0.07	0.07	0.03	0.03	0.003	0.04	0.1	0.03	0.05	0.6	0.4	0.02
	9:30 - 10:00	23	0.1	0.1	0.08	0.07	0.03	0.02	0.002	0.04	0.3	0.03	0.05	0.3	0.3	0.02
	10:00 - 10:30	20	0.1	0.09	0.07	0.09	0.03	0.03	0.003	0.05	0.3	0.04	0.05	0.2	0.5	0.02
	10:30 - 11:00	20	0.1	0.08	0.07	0.09	0.03	0.03	0.003	0.04	0.2	0.03	0.04	0.1	0.5	0.02

	11:20 - 11:50	24	0.09	0.08	0.06	0.1	0.02	0.02	0.002	0.04	0.1	0.03	0.03	0.07	0.2	0.02
	11:50 - 12:20	16	0.09	0.09	0.07	0.08	0.02	0.02	0.002	0.06	0.1	0.03	0.03	0.2	0.3	0.02
	12:20 - 12:50	20	0.1	0.08	0.06	0.09	0.02	0.03	0.003	0.05	0.2	0.03	0.03	0.2	0.4	0.02
	12:50 - 13:20	20	0.08	0.08	0.06	0.09	0.02	0.02	0.002	0.04	0.1	0.03	0.03	0.09	0.3	0.02
	13:20 - 13:50	24	0.08	0.07	0.05	0.05	0.02	0.02	0.001	0.03	0.09	0.03	0.03	0.08	0.1	0.02
	14:00 - 14:30	23	0.1	0.06	0.06	0.1	0.02	0.02	0.002	0.03	0.08	0.03	0.03	0.05	0.7	0.02
**4**	7:50 - 8:50	11	0.2	0.05	0.04	0.07	0.02	0.04	0.005	0.04	0.3	0.05	0.05	0.1	0.8	0.009
	9:50 - 10:50	9	0.1	0.06	0.04	0.08	0.02	0.02	0.002	0.04	0.5	0.02	0.04	0.2	0.3	0.009
	10:50 - 11:50	12	0.09	0.05	0.04	0.05	0.02	0.009	0.0006	0.04	0.4	0.01	0.04	0.3	0.04	0.009
	11:50 - 12:50	11	0.1	0.05	0.05	0.08	0.03	0.02	0.002	0.05	0.5	0.02	0.07	0.3	0.08	0.009
	12:50 - 13:50	11	0.2	0.07	0.1	0.1	0.04	0.04	0.005	0.2	2	0.04	0.2	2	0.1	0.01
	14:05 - 15:05	9	0.07	0.04	0.04	0.05	0.01	0.008	0.0005	0.02	0.1	0.01	0.02	0.1	0.03	0.009
**5**	7:55 - 8:25	23	0.1	0.10	0.2	0.1	0.1	0.1	0.01	0.4	1	0.03	0.08	0.3	0.09	0.02
	8:25 - 8:55	20	0.2	0.1	0.2	0.1	0.1	0.1	0.009	0.4	3	0.03	0.1	1	0.06	0.02
	8:55 - 9:25	25	0.2	0.3	4	0.2	2	6	0.7	7	10	0.8	0.2	12	0.07	0.07
	9:25 - 9:55	32	0.2	0.3	3	0.3	2	6	0.7	7	13	1	0.3	12	0.06	0.06
	9:55 - 10:25	22	0.2	0.2	0.7	0.2	0.3	1	0.1	2	14	0.2	0.5	4	0.2	0.04
	10:25 - 10:55	24	0.2	0.1	0.4	0.1	0.2	0.57	0.07	0.8	3	0.09	0.1	2	0.05	0.02
	11:25 - 11:55	21	0.3	0.2	0.6	0.6	0.6	0.63	0.07	0.8	5	0.1	0.5	2	0.3	0.03
	11:55 - 12:25	25	0.4	0.2	0.8	0.6	0.3	0.93	0.10	1	9	0.2	0.4	2	0.4	0.04
	12:25 - 12:55	21	0.2	0.1	0.3	0.4	0.2	0.262	0.03	0.4	3	0.06	0.1	0.7	0.1	0.02
	12:55 - 13:25	18	0.2	0.1	0.1	0.2	0.1	0.09	0.01	0.2	2	0.04	0.06	0.3	0.1	0.02
	13:25 - 13:55	17	0.1	0.1	0.07	0.2	0.08	0.02	0.002	0.08	1	0.03	0.04	0.3	0.05	0.02
	13:55 - 14:25	20	0.2	0.1	0.2	0.9	0.1	0.03	0.003	0.2	3	0.03	0.05	0.7	0.06	0.02

Table S3. Minimum detection limits for $PM_{2.5}$ and elemental concentrations in μg/m^3.

$PM_{2.5}$	S	Cl	K	Ca	Ti	Cr	Mn	Fe	Ni	Cu	Zn	Mo	Pb	Bi
0.8	0.2	0.1	0.07	0.05	0.04	0.04	0.03	0.04	0.03	0.03	0.03	0.1	0.8	0.06

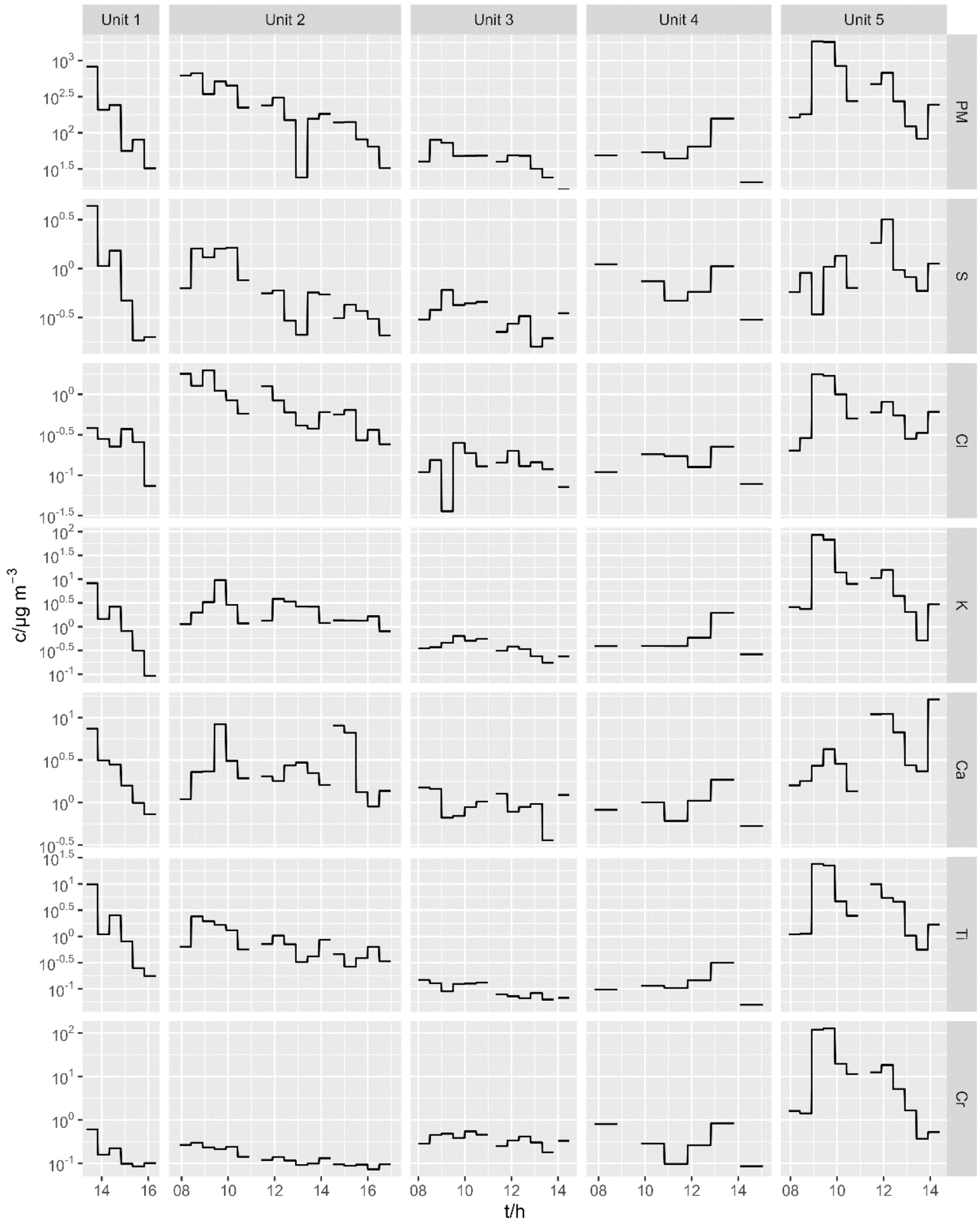

Figure S1a. Time series of $PM_{2.5}$ and elemental concentrations in five different units of a single metal workshop.

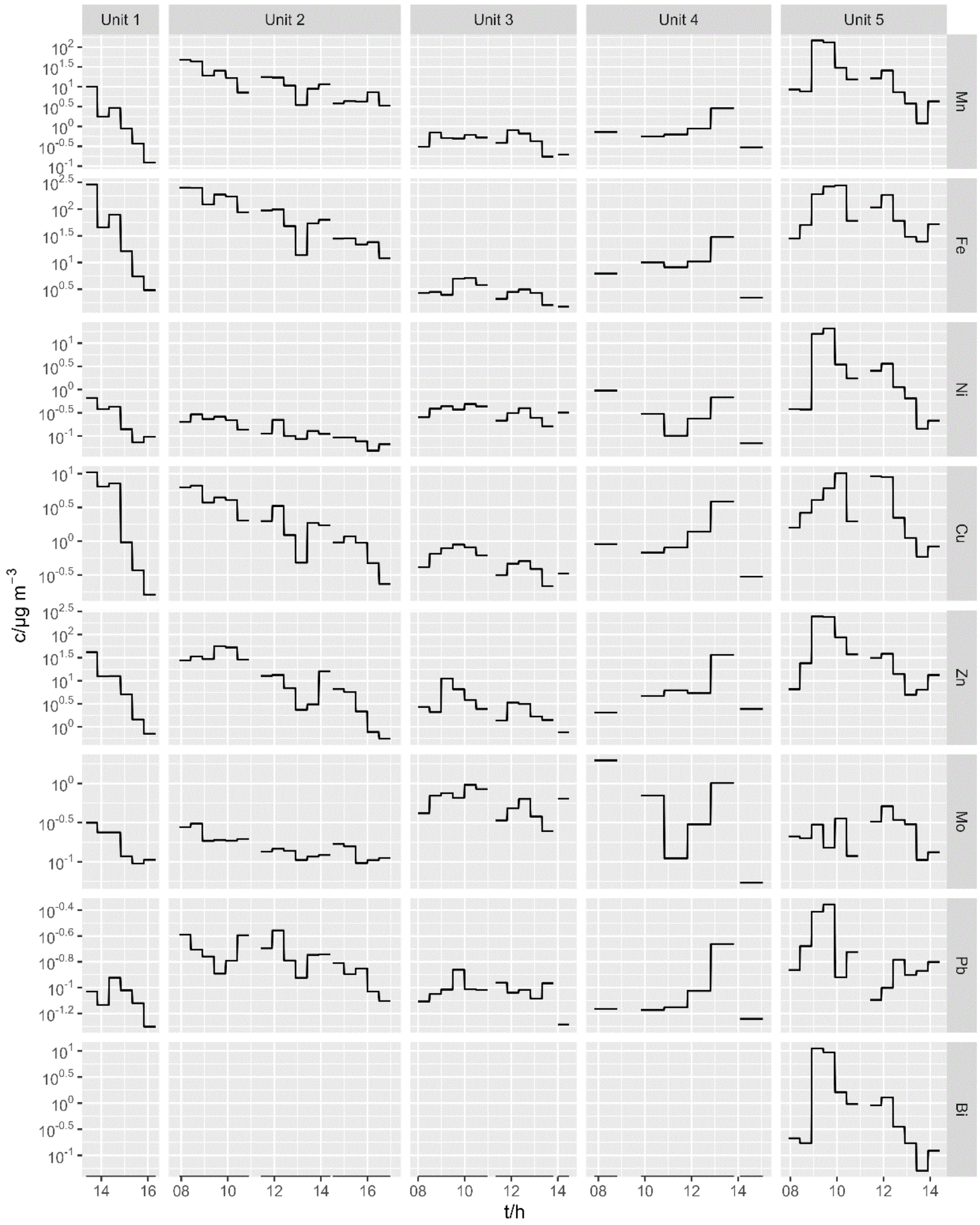

Figure S1b. Time series of elemental concentrations in five different units of a single metal workshop.
